# *Streptococcus pneumoniae* Nasopharyngeal Carriage among PCV-10-Vaccinated HIV-1-Infected Children with Maintained Serological Memory in Ethiopia

**DOI:** 10.3390/pathogens9030159

**Published:** 2020-02-25

**Authors:** Mahlet Lemma, Yonas Bekele, Stefan Petkov, Moa Hägglund, Beyene Petros, Abraham Aseffa, Rawleigh Howe, Francesca Chiodi

**Affiliations:** 1 Department of Microbiology, Tumor and Cell Biology, Biomedicum, Karolinska Institutet, Solnavägen 9, 17165 Solna, Sweden; 2 Armauer Hansen Research Institute, Jimma Road, ALERT compound P.O. Box 1005, Addis Ababa, Ethiopia; 3 Department of Microbial, Cellular and Molecular Biology, Addis Ababa University, Arat Kilo Campus, Addis Ababa, Ethiopia; 4 Science for Life Laboratory, Department of Microbiology, Tumor and Cell Biology, Karolinska Institutet, 17165 Solna, Sweden

**Keywords:** *S. pneumoniae*, PCV-10, serological memory, nasopharyngeal carriage, whole-genome sequence, HIV-1, children

## Abstract

*Streptococcus pneumoniae* (*S. pneumoniae*) vaccines have substantially reduced the burden of invasive pneumococcal diseases (IPDs) worldwide. Despite high coverage with *S. pneumoniae* vaccination, upper-respiratory-tract colonization by *S. pneumoniae* is still common. We assessed maintenance of serological responses to *S. pneumoniae* serotypes included in PCV-10 by ELISA in HIV-1-infected children (n = 50) and age-matched controls (n = 50) in Ethiopia. We isolated *S. pneumoniae* in nasopharyngeal swabs and determined *S. pneumoniae* serotype by whole genome sequencing (WGS). Comparable levels of *S. pneumoniae* serotype-specific IgG concentrations were detected in plasma of HIV-1-infected children and matched controls, with geometric mean concentrations (GMCs) consistently higher than the protective threshold for PCV-10 serotypes of 0.35 µg/mL. We isolated *S. pneumoniae* from 38 (out of 97) nasopharyngeal swabs, 25 from HIV-1-infected children and 13 from controls. WGS based serotyping revealed 22 known *S. pneumoniae* serotypes and 2 nontypeable (NT) isolates. Non-PCV-10 serotypes represented >90% of isolates. We showed that HIV-1-infected children and matched controls in Ethiopia carry a level of maintained serological memory to PCV-10 considered protective for IPDs. We identified a higher proportion of nasopharyngeal carriage with highly pathogenic *S. pneumoniae* non-PCV strains among HIV-1-infected children compared to controls.

## 1. Introduction

*Streptococcus pneumoniae* (*S. pneumoniae*), although part of the normal flora in the upper respiratory tract, can cause severe noninvasive (pneumonia, sinusitis, otitis) and invasive (bacteremia and meningitis) diseases [1,2]. Based on the variability of capsular antigens, more than 90 serotypes have been described [3]; among these are opportunistic pathogens which can cause different noninvasive and invasive pneumococcal diseases (IPD). Colonization of the upper respiratory tract is the initial step for all forms of pneumococcal disease, and the rate of colonization is higher at an early age [4]. Asymptomatic carriers can easily transmit the bacteria through respiratory droplets to healthy individuals [2]. Age, genetics, comorbidity, socio-economic status and seasons are some of the risk factors for pneumococcal diseases [5]. The risk of IPD and community-acquired pneumonia (CAP) was significantly increased during HIV-1 infection and in individuals less than 5 years and older than 65 years of age [6,7,8]. In 2017, *S. pneumoniae* was classified as one of the 12 priority pathogens [9]. 

Pneumococcal diseases are a major public health problem, particularly in low- and middle- income countries where 3.7 million invasive pneumococcal diseases were estimated to occur in 2015 with 318,000 fatal cases in children under the age of five years [10,11]. In Ethiopia, 15,000 pneumococcus related annual deaths occurred in 2015, which makes Ethiopia one of the five countries with the highest rate of pneumonia-related deceases [12]. Of the total deaths related to pneumococcal pneumonia in the African continent (137,000 cases) in 2015, approximately 10% occurred in HIV-1-infected children [10]. The details on the current impact of *S. pneumoniae* infection on IPDs in Ethiopia are missing and additional, well-designed studies are urgently needed to evaluate the burden assessment of IPDs in Ethiopia.

Pneumococcal vaccines substantially reduced the burden of IPD in HIV-1-infected individuals [13]; currently, both unconjugated (PPV) and conjugated (PCV) vaccines are available in various dosages against *S. pneumoniae* infection. The first vaccine, which has been available since 1983, is the pneumococcal polysaccharide vaccine including 23 serotypes, PPV-23 [14]. PPV-23 contains purified pneumococcal polysaccharides and has been shown to elicit protective T cell-independent B cell responses against *S. pneumoniae* in children older than two years of age and in adults [15]. Unfortunately, this polysaccharide vaccine was unable to induce protective immune responses in children under the age of two whose immune system could not yet elicit T cell-independent responses; these observations suggested the need for the development of other vaccine types for the age range 0–2 years [14,15]. Antibodies against capsular antigens are produced through short-lived humoral immunity; coupling capsular antigens with carrier proteins increased the immunogenicity and longevity of the response [16]. Marginal zone (MZ) B cells, which develop in the absence of germinal center formation, are involved in the response to *S. pneumoniae* capsular antigens; these cells, whose development is not fully completed before two years of age, can migrate and expand in response to capsular antigens and T cell-independent vaccines [17]. 

A three-dose schedule of protein-conjugated pneumococcal vaccines (PCV7/10/13) has been shown to induce protective immune responses in vaccinated children under the age of two and herd immunity in the unvaccinated community [18]. Children with IgG level of > 0.35 µg/mL are considered to have a protective antibody level towards IPDs [19]. Since these vaccines became available, a tremendous decrease in the burden of IPD caused by vaccine serotypes was observed globally. Furthermore, between 2000 and 2015 there was a 22% global decline in clinical pneumonia cases in the general children group and of 45% in the HIV-1-infected children group [12]. The 10-valent pneumococcal vaccine (PCV10) has been introduced into the national immunization program of Ethiopia with a 3-prime dose (3p+0) schedule since 2011 [20,21].

The colonization of the nasopharynx mucous membrane by *S. pneumoniae* is denominated nasopharyngeal carriage. PCVs induce an IgG response which mediates neutralizing activity during *S. pneumoniae* infection and reduces nasopharyngeal carriage by vaccine serotypes [22]. According to Wahl and collaborators [10], 27% to 65% of children and <10% of adults are carriers of *S. pneumoniae*. CD4+ T cells, through an antibody-independent mechanism, have also been shown to play a role in mediating protection against *S. pneumoniae* colonization in experimental models and exposed individuals, including children [23,24]. Two distinct CD4+ T cell lineages, Th1 and Th17, facilitate clearance of *S. pneumoniae* carriage within the nasopharynx; CD4+ T cell depletion during HIV-1 infection has been associated with defects within the Th1 T cell compartment [25], thus compromising *S. pneumoniae* clearance in symptomatic HIV-1-infected individuals. In this context, colonization rate in HIV-infected Malawian adults remained high following anti-retroviral treatment (ART) for 12 months [26]. Viral coinfection [27], close contact and young age have been reported to play a major role in *S. pneumoniae* transmission. In the era of PCVs, there is a replacement of vaccine serotypes by nonvaccine serotypes in nasopharyngeal carriage [20,21,28].

Healthy individuals have been reported to exhibit higher *S. pneumoniae* serotype-specific IgG and IgM levels compared to HIV-1-infected individuals up to one year postvaccination with PPV-23 and PCV vaccines [29,30]. In contrast, other studies reported a comparable level of specific IgG production in both HIV-1-infected and -uninfected individuals after PCV vaccination, although with a significant difference in the effectiveness of the produced antibodies against IPD [31,32]. Nasopharyngeal carriage of nonvaccine serotypes is still a common phenomenon in PCV-vaccinated HIV-1-infected children in Africa [33]. In this study, we assessed the maintenance of humoral responses to PCV-10 in HIV-1-infected children and age-matched controls and studied nasopharyngeal carriage in these individuals by determining the serotype of *S. pneumoniae* in nasopharyngeal swabs with whole-genome sequencing (WGS). 

## 2. Results

### 2.1. S. pneumoniae Serotype-Specific Antibody Responses 

Persistence of *S. pneumoniae* serotype-specific serological memory was detected in HIV-1-infected children and age-matched controls after a median time period of 4.9 years from PCV-10 vaccination; the clinical characteristics of patients and controls included in the study are presented in Table 1.

**Figure 1:**
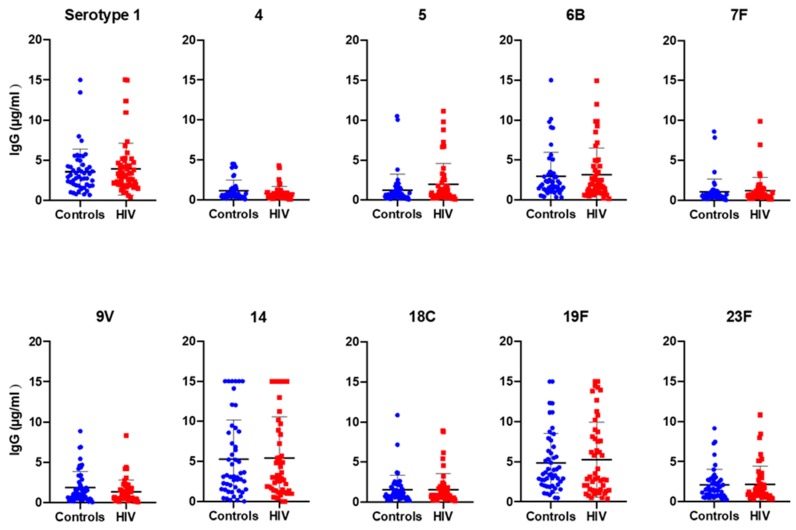
Median level of IgG concentration against the 10 *S.* *pneumoniae* serotypes included in PCV-10. Plasma samples from 50 control and 50 HIV-1-infected children were studied for their reactivity to PCV-10 serotype-specific IgGs.

A comparable level of serotype-specific IgG concentrations was detected in both HIV-1-infected children and age-matched controls (Figure 1). 

The IgG geometric mean concentration (GMC) to all serotypes incorporated in PCV-10 was shown to be consistently higher than the conventional threshold of 0.35 µg/mL, which is the protective threshold antibody level for IPD (Table 2), with the highest IgG GMC detected for serotype 19F (3.59 µg/mL in controls and 3.36 µg/mL in HIV-1-infected children) and the lowest for serotype 7F (0.63 µg/mL in controls) and serotype 4 (0.56 µg/mL in HIV-1-infected children). 

The percentage of individuals with antibody level ≥ 0.35 µg/mL lies between 75% (serotype 4) and 100% (serotype 1 and 19F; Table 2), with all other serotypes in between these two values. There was no statistically significant difference between the GMCs of the two studied groups and the percentage of individuals with ≥ 0.35µg/mL for the 10 serotypes. However, a lower percentage of individuals with ≥ 0.35 µg/mL was detected for serotype 7F (76%) in the controls and serotype 4 (68%) in the HIV-1-infected group compared to the other serotypes (> 80% of protected individuals) (Table 2). A high proportion of individuals (58% HIV-1-infected and 60% controls) had protective antibody levels to all 10 serotypes, and 2% of children from each group showed a protective level of antibody only for 3 serotypes included in the vaccine (results not shown).

**Figure 2:**
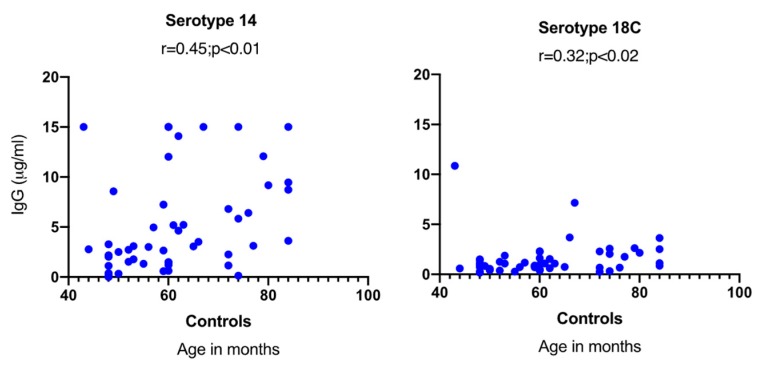
Correlation between age and serotype-specific IgG concentration in control children. A significant correlation was only noticed between age and specific IgG concentrations in control children for serotypes 14 and 18C.

A significant positive correlation was observed between the age of the controls and serotype-specific IgG concentrations for serotype 14 (*p* < 0.01) and 18C (*p* < 0.05) (Figure 2). In HIV-1-infected children, on the other hand, no significant correlation was found between age and serotype-specific IgG concentration, and no differences in GMCs were observed between viremic and aviremic children (data not shown).

### 2.2. Determination of S. pneumoniae Nasopharyngeal Carriage with Whole-Genome-Sequencing-Based Serotyping and Multi-Locus Sequence Typing (MLST) 

Pneumococcal carriage was detected in 38 out of 97 (39.1%) nasopharyngeal swabs collected from PCV-10-vaccinated children, 25 HIV-1-infected children and 13 healthy controls. A statistically significantly higher carriage rate of *S. pneumoniae* was detected in HIV-1-infected children (52%) compared to age-matched healthy controls (26.5%) (*p* = 0.01). 

**Figure 3:**
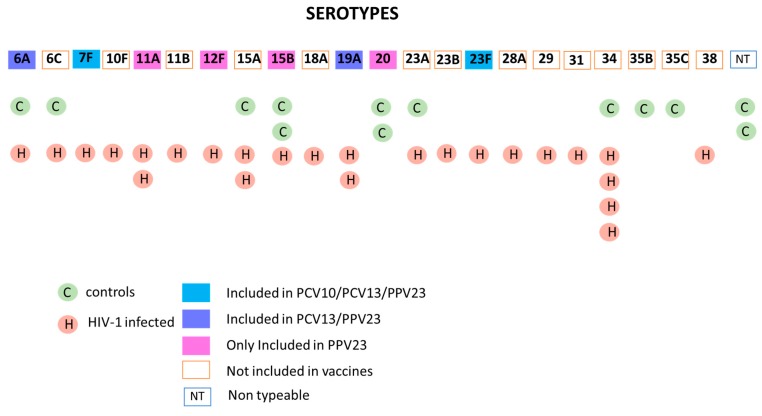
*S. pneumoniae* serotypes present in nasopharyngeal swabs of PCV-10-vaccinated HIV-1-infected and control children. The figure shows the serotype of *S. pneumoniae* isolates obtained from nasopharyngeal swabs of HIV-1-infected children (25 isolates) and age-matched controls (13 isolates) derived through whole-genome sequencing (WGS). The boxes filled with the different colors indicate the isolates included in the different *S. pneumoniae* vaccines; empty boxes represent the serotypes not included in the vaccines or nontypeable.

Results from WGS-based pneumococcal serotyping showed that 36 *S. pneumoniae* isolates comprised 22 known serotypes and 2 isolates classified as nontypeable (NT). Among the isolates, non-PCV-10 serotypes represented more than 90% of the serotypes characterized (Figure 3). In addition to nonvaccine serotypes, two serotypes (8.7%) are included in PCV-10 (7F and 23F), two (8.7%; 6A and 19A) in PCV-13 and four (17.4%; 11A; 12F, 15B, 20) in PPV23.

A higher percentage of vaccine serotypes were detected in HIV-1-infected children compared to age-matched healthy controls (Figure 3). PCV-10 serotypes are responsible for 8% of the carriage in HIV-1-infected children (which included a total of 25 isolates) and were not detected in the serotypes isolated (n = 13) from age-matched controls. PCV-13 serotypes 6A and 19A contributed 12% and 7.6% of pneumococcal carriage in HIV-1-infected children and controls, respectively. On the other hand, the carriage rate of vaccine serotypes incorporated in PPV23 is two times higher in controls (31%) than in HIV-1-infected children (16%). Despite the higher rate of PCVs serotypes carriage in HIV-1-infected children with history of PCV-10, 80% of carriage was caused by nonvaccine serotypes which are not incorporated in either PCV10 or PCV13.

Serotype 34 was the most common serotype isolated from the nasopharyngeal swabs (13.1%). Serotypes 15A and 15B each contribute to 7.8% of the nasopharyngeal carriage isolates followed by 6A, 6C, 19A, 20 and 23A (5.2% each), as shown in Figure 3. There was no statistically significant difference in the GMCs against *S. pneumoniae* serotypes between children with nasopharyngeal carriage and noncarriers for both groups except for serotype 14 in controls, where the noncarriers had a higher GMC than the carriers (*p* = 0.01).

**Figure 4:**
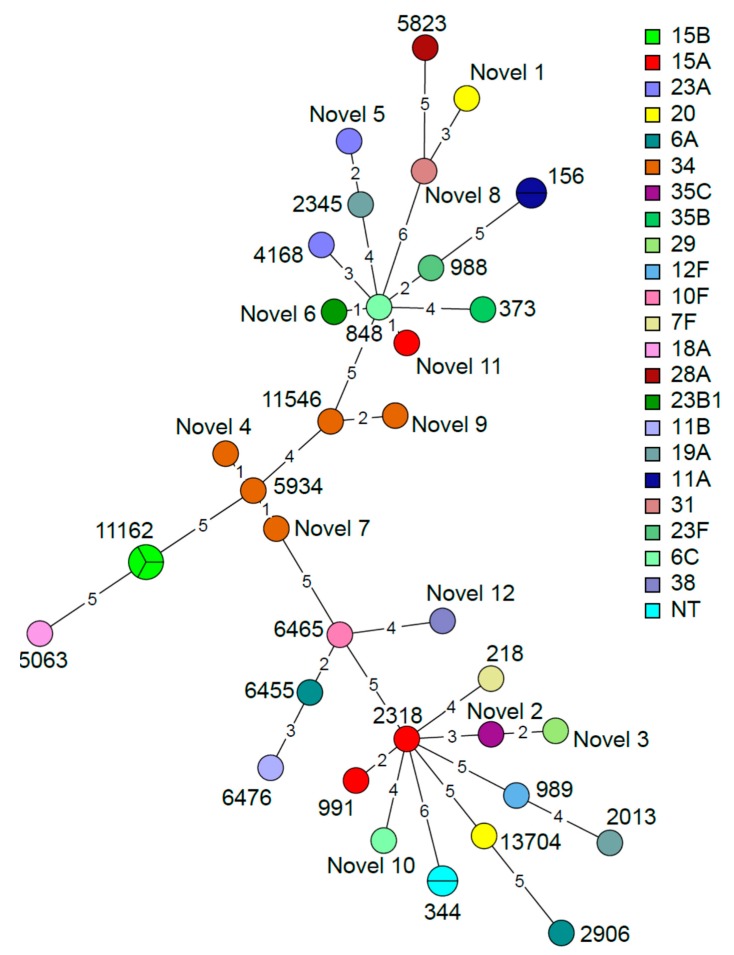
Minimum spanning tree based on the multi-locus sequence typing (MLST) results of the 38 S. pneumoniae isolates grouped by sequence type. The nodes are colored according to serotype. The size and partition of the nodes indicate how many samples are included in each node. The branch length indicates the number of alleles that differ between the sequence types.

Using multi-locus sequence typing (MLST) analysis (Table 3), which, through sequencing, identifies seven alleles in the *S. pneumoniae* sequences, 34 *S. pneumoniae* sequence types (ST) were identified; among these, 12 are novel types, suggesting that *S. pneumoniae* strains from Ethiopia may be underrepresented in the MLST database. ST11162 is the most frequent sequence type, followed by ST344 and ST156. In addition, various sequence types were also comprised in the same serotype: serotype 34 (ST11546, ST5934 and three novel STs), serotype 15A (ST991, ST2318 and one novel ST), serotype 23A (ST4168 and one novel ST) and serotype 20 (ST13704 and one novel ST). A graphical representation of the MLST findings is presented in Figure 4 showing a network tree where the phylogenetic relationship of the alleles characterized from the different *S. pneumoniae* isolates is shown. As shown in Figure 4, some novel STs are single- or double-loci variants, but for other STs, several loci variations were identified.

### 2.3. S. pneumoniae Serotype-Specific Antibody Responses in Relation to Nasopharyngeal Carriage

It has been previously suggested that protection from *S. pneumoniae* carriage should be evaluated considering different IgG thresholds for the different serotypes included in PCV-10 or PCV-13 [35]. We took into consideration these different thresholds and calculated the frequency of individuals who showed levels of protective antibody responses against carriage with the different serotypes. The results are presented in Table 4. The largest frequency of individuals showing protective levels was detected for serotypes 1 (94%) and 6B (93%) and the lowest for serotypes 4 (26%) and 7F (13%), and these values were comparable in both groups of healthy controls and HIV-1-infected children. These results should be taken into consideration when evaluating the complex picture of immunological interactions ensuring protection against *S. pneumoniae* carriage. It is interesting and puzzling that the two HIV-1-infected children displaying nasopharyngeal carriage for PCV-10 serotypes 7F and 23F had protective levels against the two serotypes substantially above (9.89 and 2.1 µg/mL, respectively) the suggested threshold value suggested for 7F and 23F serotypes in plasma. 

## 3. Discussion

This study is the first study to report, in Ethiopia, the maintenance of *S. pneumoniae* serotype-specific antibody responses in ART-treated HIV-1-infected children and age-matched controls previously vaccinated with PCV-10. The children’s median age was 4.9 years, and they had all received a full dose of PCV-10 at 6, 10 and 14 weeks of age. In the study, similar percentages of HIV-1-infected children and age-matched controls were found to carry antibody concentrations considered to be protective against IPD (≥0.35 µg/mL) for all serotypes included in PCV-10. A study conducted by Madhi and collaborators [36] reported a similar result among HIV-1-infected and noninfected children who had received PCV10 (three doses plus one booster) with samples collected 14 months after the booster. Furthermore, the persistence of serotype-specific IgG responses in non-HIV-1-infected children reported by other studies after 3, 4 and 5 years of completed PCV-10 vaccination is comparable with our results [37,38,39]. These findings confirm the ability of PCV-10 to induce a long-lasting serotype-specific IgG response. The maintenance of high protective levels of *S. pneumoniae* antibodies in plasma after two to six years of vaccination may, on the other hand, also reflect natural exposure to *S. pneumoniae* serotypes included in PCV10, which may reinvigorate immunological memory elicited during vaccination. 

It has previously been reported that, in the current era of PCVs, there is a replacement of vaccine by nonvaccine *S. pneumoniae* serotypes, with the latter isolated from nasopharyngeal carriage and IPD patients, particularly in individuals with underlying conditions including HIV-1 infection [20,21,28,40]. In this study, nasopharyngeal carriage was detected in 39.1% of children who had received the full dose of PCV-10, with over 90% of the carriage caused by nonvaccine serotypes not included in PCV-10. The carriage rate in the vaccinated children included in the present study is higher than that previously reported in Ethiopia (21.5%) [20].

In contrast to the comparable amount of specific plasma IgG levels to the serotypes included in PCV-10, we detected that the carriage rate in HIV-1-infected children (52%) was twice the carriage rate in age-matched controls (26.5%); carriage of vaccine serotypes 7F and 23F was only detected in the HIV-1-infected group. This result indicates the need for immunological studies assessing the mechanisms of immune responses against pneumococcal nasopharyngeal carriage in PCV-10-vaccinated individuals, particularly in HIV-1-infected children, in areas with a high burden of pneumococcal diseases. Apart from 7F and 23F, nasopharyngeal carriage by other serotypes included in PCV-10, serotypes 1, 4, 14 and 19F, was previously reported in Ethiopia [20,21]. PCV13 serotypes 6A and 19A each contributed 5.2% of pneumococcal carriage in our study; this result is significantly lower than what previously shown by Negash et al. [20] for 19A (27%) but comparable to what was shown for 6A (4.9%) in children with CAP after 5 years of PCV-10 introduction. All *S. pneumoniae* serotypes reported in this study, except 28A, were previously reported in nasopharyngeal carriers in Ethiopia [20,21]. The MLST analyses, which characterize microorganisms by their allelic profiles, suggested that 12 of the 38 *S. pneumoniae* isolates in our study carry novel combinations of alleles; a large scale study should be performed in Ethiopia to further characterize the genomic profile of *S. pneumoniae* and the novelty degree of the alleles identified by us. Most carriage serotypes isolated and characterized in our study have previously been associated with complications of *S. pneumoniae* infection, from otitis media to IPD cases, including pneumococcal meningitis, in different parts of the world [40,41,42,43]. Therefore, our findings on the carriage in HIV-1-infected children are worrisome as these individuals, despite ART treatment and PCV-10 vaccination, remain at high risk for *S. pneumoniae*-related complications. 

The immunological mechanisms responsible for protection against *S. pneumoniae* carriage remain elusive, and arguments have previously been presented for innate and adaptive immune responses playing a role in this context. MZ B cells, which are found in the spleen MZ [44] and to some extent also in blood, play an important role in T-cell-independent humoral immunity to encapsulated bacteria rich in polysaccharides antigens, including *S. pneumoniae* [45]. MZ B cells are fully developed in humans around two years of age [46]. A decline in frequency of mature MZ B cells has been reported in the blood of HIV-1-infected children, probably due to impaired migratory capacity of these cells [47]. HIV-1 infection leads to the dysregulation of several B cell subpopulations, including memory B cells [48,49], which, together with MZ B cells, are associated with maintenance of serological memory to PCV vaccination. Inflammation and impaired control of human innate immune responses have recently emerged as important pathogenic players in establishment of *S. pneumoniae* infection and associated clinical conditions [50]; respiratory viruses may play a negative role in carriage establishment by leading to increased inflammation. 

It is still unclear whether protection from nasopharyngeal colonization by vaccine serotypes is due to IgG and/or IgA produced locally or by the leakage of circulating IgG into the mucosa. Despite the observation that PCVs induce comparable amounts of serotype-specific *S. pneumoniae* antibodies in HIV-1-infected and noninfected individuals, the functional capacity of these antibodies to neutralize *S. pneumoniae*, as measured by opsonophagocytic assay, was lower in the HIV-1-infected group [51,52]. According to an analysis conducted by Voysey M. et al. [35], the antibody concentration required for protection against pneumococcal carriage varies for each serotype, and a higher antibody concentration is needed for carriage protection in developing countries. In our study, there was no significant difference in the *S. pneumoniae* serotype-specific antibody concentration between HIV-1-infected and control children using the reference reported by Voysey M. et al. [35]. Voysey M. [35] also suggested that pneumococcal antibody thresholds of protection may be different in low- and high-income countries [35]; further studies conducted in African countries may, however, be needed to create reference values for protection against pneumococcal carriage in this continent. The balance between Th17 and Treg cells in nasopharynx-associated lymphoid tissue has been reported to play a role for clearance of *S. pneumoniae* in this compartment; Th17 cell frequencies increase with age [53]. The lack of knowledge on the CD4+ T cell counts is a limitation of the present study. Longitudinal studies in HIV-1-infected and noninfected children, regularly assessing memory and MZ B cells before and after PCV-10 vaccination in parallel with determination of T cells and antibody responses to *S. pneumoniae* serotypes and carriage, may lead to significant information on the relation between cellular and serological components relevant to protection from *S. pneumoniae* infection and carriage.

## 4. Material and Methods 

### 4.1. Study Population

A cross-sectional study was conducted between May 2018 and March 2019, which included 50 ART-treated HIV-1-infected children (median age in months and range 53.5 (24–89)) and 50 age-matched controls (age and range 60 (43–84)) who had received 3 primary doses of 10-valent pneumococcal vaccine (PCV-10) according to the Ethiopian childhood immunization program. Vaccination history was confirmed using a vaccination history record. All HIV-1-infected children were recruited from ALERT Hospital, Zewditu Memorial Hospital and Yekatit 12 Hospital ART clinics and control subjects from Woreda 03 Health Center, Addis Ababa. 

HIV-1 RNA copies were quantified in plasma of HIV-1-infected children using an automated m2000sp Abbott Real-Time HIV-1 assay system following the manufacturer’s protocol (Abbott Laboratories, Abbott Park, IL, USA) (Table 1). The lower detection limit of this assay was 40 copies/mL. Viremia was detected in 40% of HIV-1-infected children with a median viral load of 42,095 copies/mL (range 266–1,882,592). There was no correlation between the viremic status, age and length of treatment.

Blood samples were collected from all study participants; plasma was isolated and stored at −80 °C. Nasopharyngeal swab specimens (n = 97) were obtained using a flexible calcium alginate-tipped swab which was stored in skim milk–tryptone–glucose glycerin medium (STGG) at −80 °C. Samples were transported frozen to Karolinska Institutet for further analyses. 

### 4.2. Measurement of S. pneumoniae Serotype Specific IgG in Plasma

The plasma concentration of IgG specific for the 10 individual *S. pneumoniae* serotypes included in PCV-10 was measured by ELISA using purified pneumococcal polysaccharide antigens donated from SSI Diagnostica (Hillerød, Denmark) and 007SP as a standard reference serum (gift from Dr. Mustafa Akkoyunlu, FDA, USA). The WHO guideline for pneumococcal ELISA was followed with few modifications [54]. Briefly, ELISA plates were coated using 5 µg/mL of purified pneumococcal polysaccharide. The coated plates were incubated for 5 h at 37 °C and stored overnight at +4 °C. Thereafter, they were washed using 0.05% tween in PBS and blocked using 1% BSA. To avoid nonspecific binding, standard reference serum and serum samples were adsorbed using an adsorption solution which contains 10 µg/mL of pneumococcal cell wall polysaccharide and *S. pneumoniae* serotype 22F for 1 h at 37 °C. Following adsorption, 100 µL of standard and serum samples were added to the plates in duplicate. After 2 h of incubation, 100 µL of anti-human IgG secondary antibody conjugated with alkaline phosphatase were added, followed by substrate (p-nitrophenyl phosphate) and 3M NaOH stopping solution after 1 h incubation at room temperature. All samples and standards were tested in duplicate. Interpolation of concentration for the samples was done in GraphPad Prism 8. Children with IgG level of ≥ 0.35 µg/mL are considered to have IPD-protective antibody levels [19].

### 4.3. Identification of S. pneumoniae from Nasopharynx Swabs and DNA Isolation

Culture-based identification of *S. pneumoniae* from nasopharyngeal swabs was conducted using the 2016 CDC guidelines [55]. Each swab was plated into trypticase soy agar (TSA) plates supplemented with 5% sheep blood containing 5 µg/mL gentamicin. Identification of *S. pneumoniae* was performed based on colony morphology and Optochin susceptibility. Pure α-hemolytic optochin susceptible *S. pneumoniae* isolates were stored at −80 °C in STGG. QIAamp® BiOstic® Bacteremia DNA Kit (Qiagen, Hilden, Germany) was used for DNA isolation. 

### 4.4. Whole Genome Sequence-Based S. pneumoniae Serotyping 

Whole-genome sequencing was conducted at Scilife Laboratory (Stockholm, Sweden). Genomic DNA was quantified using Quant-iT^TM^ dsDNA High-Sensitivity Assay Kit and FLUOstar Omega plate reader. Samples were normalized to 2 ng/µL; 1 µL of normalized genomic DNA was used in the tagmentation reaction using Nextera chemistry (Illumina, San Diego, CA, USA) to yield fragments of approximately 410 bp. Amplification of tagmented library was carried out using 11 cycles of PCR with Unique Dual Index (UDI) (IDT Technologies, Coralville, IA, USA). The resulting PCR products were purified using Sera-Mag beads (GE Healthcare Life Sciences, Uppsala Sweden). The obtained library was quantified using Quant-iT^TM^ dsDNA High-Sensitivity Assay Kit and FLUOstar Omega plate reader. Library was pair-end (2x151 bp) sequenced to minimum 3 M read pairs on NovaSeq 6000 (Illumina, San Diego, CA, USA). Base-calling and demultiplexing was done using bcl2fastq v2.20.0.422, allowing one mismatch in the index sequence.

The microbial sequence analysis and loci-based typing pipeline MicroSALT v. 2.8.9 (https://github.com/Clinical-Genomics/microSALT) was utilized to analyze the sequence data. MicroSALT is an open-source pipeline that is based on publicly available software. The software that are included in the pipeline are BLAST v. 2.9.0, BWA v. 0.7.17 [56], Picard tools v 2.20.3 (https://github.com/broadinstitute/picard), QUAST v. 5.0.2 [57], SAMtools v 1.9 [58], SPAdes v. 3.13.1 [59] and Trimmomatic v. 0.39 [60]. MicroSALT utilizes the databases PubMLST and ResFinder. BLAST was utilized to search in PubMLST to assign sequence type loci to the assembled sequences. 

The serotypes of the strains were predicted using SeroBA v. 1.0.1 [34]. The k-mer counts database was built through SeroBA using the recommended setting for the kmer size (71 bp). Default settings were utilized for the analysis except that, in order to include all serotypes in the ARIBA-compatible database, the maximum allowed length in nucleotides of non-coding sequences was set to 25,000 bp.

The results of the MLST and serotype analyses are visualized in a minimum spanning tree using the BioNumerics software (Version 7.6, Applied Maths NV, Sint-Martens-Latem, Belgium).

### 4.5. Statistical Analysis 

Demographic and clinical data were analyzed using Graph pad prism 8 (La Jolla, CA, USA). The normal distribution of ELISA data was assessed using Kolmogorov–Smirnov test. Unpaired T-test was used to assess the difference between groups. Fisher exact test was used to compare frequency between groups. Spearman correlation was applied to determine the relation of variables. A *p*-value < 0.05 was considered statistically significant. 

### 4.6. Ethical Statement 

Ethical clearance to conduct the current study was obtained from the ethical clearance committee of ALERT/AHRI ethics review committee (protocol number P0/06/17) and Ethiopian national ethical review committee (NERC; reference n. 3.10/71/2018). Informed written consent was collected from parents or legal guardians of children in the study. The study was conducted in accordance with the Declaration of Helsinki. The ethical committee at Karolinska Institutet approved the laboratory studies of the collected specimens (approval n 2016/485-32). 

## Figures and Tables

**Table 1:** Demographic and clinical data of the study participants.

Characteristics	PCV-10-Vaccinated	
	Controls (n = 50)	HIV-1-Infected Children (n = 50)
Median age (range) in months	60 (43–84)	53.5 (24–89)
Gender		
Female	25 (50%)	27 (54%)
Male	25 (50%)	23 (46%)
Viral load *		
Aviremic	NA	26 (52%)
Viremic	NA	20 (40%)
Median viral load	NA	42,095 (266–1,882,592)
ART		
ABC + 3TC + NVP	NA	10.3%
AZT + 3TC + NVP	NA	30.7%
AZT + 3TC + EFV	NA	10.3%
AZT + 3TC + LPV/r	NA	23.1%
ABC + 3TC-LPV/r	NA	25.6%
Months on ART: median (range)	NA	40 (11–83)
WHO stage *		
Stage I	NA	46
Stage III	NA	1
Body mass index	15.64 (12.42–19.78)	14.49 (8.41–36.98)

NA = not applicable. * = viral load was missing for four HIV-1-infected children and the WHO classification for three children. AZT = Zidovudine; 3TC = Lamivudine; NVP = Nevirapine; EFV = Efavirenz; ABC = Abacavir; LPV/r = Lopinavir/Ritonavir.

**Table 2:** Geometric mean concentration (GMC) of IgG to *S. pneumoniae* serotypes and number of HIV-1-infected and control individuals with specific IgG concentration ≥ 0.35 µg/mL.

Serotypes PCV-10	GMC * in Controls µg/mL	GMC in HIV µg/mL	*p*-Value	Controls N (%)	HIV N (%)	*p*-Value	Total%
1	2.84	2.9	0.73	50 (100)	50 (100)	>0.99	100
4	0.71	0.56	0.19	41 (82)	34 (68)	0.16	75
5	0.68	1	0.09	40 (80)	41 (82)	>0.99	81
6B	1.9	1.9	0.99	49 (98)	48 (96)	>0.99	97
7F	0.63	0.72	0.44	38 (76)	42 (84)	0.45	80
9V	1.04	0.82	0.28	44 (88)	42 (84)	0.77	86
14	2.78	2.59	0.92	47 (94)	48 (96)	>0.99	95
18C	1.03	0.98	0.76	45 (90)	44 (88)	>0.99	89
19F	3.59	3.36	0.70	50 (100)	50 (100)	>0.99	100
23F	1.52	1.4	0.67	48 (96)	50 (100)	0.49	98

* The IgG GMC was calculated using log-transformed IgG concentration for all serotypes included in PCV-10.

**Table 3:** Genetic characteristics of *S. pneumoniae* isolates from nasopharyngeal carriage.

Sample ID	Age (Months)	Allele No.#							MLST Sequence Type#	Serotype¤
		*aroE*	*gdh*	*gki*	*recP*	*spi*	*xpt*	*ddl*		
C12	50	7	13	8	5	9	6	8	848	6C
C15	49	6	28	4	5	17	20	148	11162	15B
C16	72	6	28	4	5	17	20	148	11162	15B
C19	60	8	5	4	1	6	1	6	2318	15A
C21	84	7	13	8	6	15	2	8	4168	23A
C26	63	1	16	4	17	7	1	14	13704	20
C29	53	8	37	9	29	2	12	53	344	NT
C30	52	8	37	9	29	2	12	53	344	NT
C33	52	1	2	29	4	43	277	255	Novel 1	20
C48	48	6	57	83	28	7	1	15	2906	6A
C49	60	162	13	4	10	17	115	28	5934	34
C54	48	8	5	36	3	6	1	17	Novel 2	35C
C67	74	7	13	4	5	7	88	9	373	35B
HIV02	53	8	5	36	3	15	1	6	Novel 3	29
HIV04	82	12	5	89	8	6	112	14	989	12F
HIV05	70	2	42	2	1	6	115	20	6465	10F
HIV08	48	2	42	9	1	6	19	20	6455	6A
HIV12	75	162	13	196	10	17	115	28	Novel 4	34
HIV18	40	10	20	14	1	6	1	29	218	7F
HIV20	61	2	16	8	6	25	6	28	Novel 5	23A
HIV21	76	25	31	4	5	32	28	44	5063	18A
HIV22	95	1	32	227	1	15	26	11	5823	28A
HIV23	76	7	13	8	5	3	6	8	Novel 6	23B1
HIV26	43	2	12	9	1	6	20	5	6476	11B
HIV27	44	2	13	8	6	25	6	14	2345	19A
HIV30	40	7	11	10	1	6	8	1	156	11A
HIV31	89	162	42	4	10	17	115	28	Novel 7	34
HIV32	75	1	2	29	1	43	7	8	Novel 8	31
HIV33	26	12	19	36	17	6	20	14	2013	19A
HIV34	31	6	28	4	5	17	20	148	11162	15B
HIV35	50	7	11	10	1	6	8	1	156	11A
HIV38	84	7	13	8	5	6	6	124	988	23F
HIV40	24	10	34	4	10	495	405	75	Novel 9	34
HIV41	81	10	13	4	10	495	405	8	11546	34
HIV42	49	54	5	4	1	6	1	111	991	15A
HIV55	52	58	5	4	1	218	79	5	Novel 10	6C
HIV76	34	7	5	8	5	9	6	8	Novel 11	15A
HIV77	83	2	555	673	1	6	83	31	Novel 12	38

**# =** The microbial sequence analysis and loci-based typing pipeline MicroSALT v. 2.8.9 (https://github.com/Clinical-Genomics/microSALT) was utilized to analyze the sequence data. ¤ = The serotypes of the strains were predicted using SeroBA v. 1.0.1 [34].

**Table 4:** Percentage of HIV-1-infected and control children with *S. pneumoniae* serotype-specific IgG concentration considered protective for *S. pneumoniae* carriage.

Serotypes in PCV-10	Antibody Threshold * (µg/mL)	Positive Controls N (%)	Positive HIV-1 Infected N (%)	*p*-Value	% of Total Children
1	0.81	46 (92%)	48 (96%)	0.68	94%
4	1.16	15 (30%)	11 (22%)	0.49	26%
5	0.73	23 (46%)	30 (60%)	0.22	53%
6B	0.5	46 (92%)	47 (94%)	>0.99	93%
7F	1.6	5 (10%)	8 (16%)	0.55	13%
9V	1.31	22 (44%)	20 (40%)	0.84	42%
14	2.48	32 (64%)	30 (60%)	0.84	62%
18C	1.32	18 (36%)	18 (36%)	>0.99	36%
19F	2.54	35 (70%)	30 (60%)	0.40	65%
23F	0.63	42 (84%)	41 (82%)	>0.99	83%

*The threshold level of serotype-specific IgG considered to be protective for nasopharyngeal carriage of PCV-10 serotypes [35] was used to calculate the percentage of individuals above IgG threshold levels.
